# Gold nanoparticles administration induced prominent inflammatory, central vein intima disruption, fatty change and Kupffer cells hyperplasia

**DOI:** 10.1186/1476-511X-10-133

**Published:** 2011-08-05

**Authors:** Mohamed Anwar K Abdelhalim, Bashir M Jarrar

**Affiliations:** 1 Department of Physics and Astronomy, College of Science, King Saud University, Saudi Arabia; 2 College of Applied Medical Sciences, Al-Jouf University, P.O. Box (2014), Skaka - Al-Jouf, Saudi Arabia

**Keywords:** gold nanoparticles, size, hepatic tissue, histology, inflammatory, fatty changes, nanotoxicity, rats

## Abstract

**Background:**

Advances in nanotechnology have identified promising candidates for many biological, biomedical and biomedicine applications. They are being increasingly exploited for medical uses and other industrial applications. The aim of the present study was to investigate the effects of administration of gold nanoparticles (GNPs) on inflammatory cells infiltration, central vein intima disruption, fatty change, and Kupffer cells hyperplasia in the hepatic tissue in an attempt to cover and understand the toxicity and the potential threat of their therapeutic and diagnostic use.

**Methods:**

A total of 70 healthy male Wistar-Kyoto rats were exposed to GNPs received 50 or 100 μl of GNPs infusion of 10, 20 and 50 nm GNPs for 3 or 7 days. Animals were randomly divided into groups, 12 GNPs-treated rats groups and one control group (NG). Groups 1, 2 and 3 received infusion of 50 μl GNPs of size 10 nm (3 or 7 days), size 20 nm (3 or 7 days) and 50 nm (3 or 7 days), respectively; while groups 4, 5 and 6 received infusion of 100 μl GNPs of size 10 nm, size 20 nm and 50 nm, respectively.

**Results:**

In comparison with respective control rats, exposure to GNPs doses has produced alterations in the hepatocytes, portal triads and sinusoids. The alterations in the hepatocytes were mainly vacuolar to hydropic degeneration, cytopasmic hyaline vacuolation, polymorphism, binucleation, karyopyknosis, karyolysis, karyorrhexis and necrosis. In addition, inflammatory cell infiltration, Kupffer cells hyperplasia, central veins intima disruption, hepatic strands dilatation and occasional fatty change together with a loss of normal architechiture of hepatic strands were also seen.

**Conclusions:**

The alterations induced by the administration of GNPs were size-dependent with smaller ones induced more affects and related with time exposure of GNPs. These alterations might be an indication of injured hepatocytes due to GNPs toxicity that became unable to deal with the accumulated residues resulting from metabolic and structural disturbances caused by these NPs. These histological alterations may suggest that GNPs interact with proteins and enzymes of the hepatic tissue interfering with the antioxidant defense mechanism and leading to reactive oxygen species (ROS) generation which in turn may induce stress in the hepatocytes to undergo necrosis.

## Introduction

In vivo studies in rats exposed to aerosols of GNPs revealed that the NPs were rapidly taken into the system with the highest accumulation in the lungs, aorta, esophagus and olfactory bulb [1]. Moreover, particles of nano-dimension are believed to be more biologically reactive than their bulk counter parts due to their small size and larger surface area to volume ratio [1,2].

Gold in its bulk form has long been considered an inert, noble metal with some therapeutic and even medicinal value hence GNPs are thought also to be relatively non-cytotoxic [3]. Yet there are differing reports of the extent of the toxic nature of these particles owing to the different modifications of the GNPs, surface functional attachments and shape and diameter size of the nanospheres [4,5]. Moreover, the metallic nature of the metal derived NPs and the presence of transition metals encourages the production of reactive oxygen species (ROS) leading to oxidative stress [6,7].

Although some scientists consider NPs as nontoxic [8-10], other cellular mechanisms such as cell signaling and other normal cellular functions may be disrupted and are currently undergoing further investigation [11,12]. The toxicity of NPs is being addressed by a number of standardized approaches with in vitro, in vivo as well as detailed genomic or biodistribution studies [12].

It has been shown that NPs may produce in vitro toxicity in some cell-based assays, but not in others. This may be a result of interference with the chemical probes, differences in the innate response of particular cell types, or other factors [13]. In addition, GNPs are used as carriers for the delivery of drugs and genes [14].

GNPs can easily enter cells and the demonstration that amine and thiol groups bind strongly to GNP has enabled their surface modification with amino acids and proteins for biomedical applications [15,16].

The histological and histochemical characterization in the hepatic tissues due to GNPs has not been documented and identified. In the present study, an attempt has been made to characterize the possible histological alterations in the hepatic tissues after the administration of GNPs and, if so, whether are related to the size of these GNPs and the time of exposure.

The present study was carried out to investigate particle-size and administration period effects of GNPs on inflammatory, central vein intima, fatty and Kupffer cells hyperplasia disruption in an attempt to cover and understand the toxicity and potential threat of their therapeutic and diagnostic use.

## Materials and methods

A total of 70 healthy male Wistar-Kyoto rats obtained from the Laboratory Animal Center (College of Pharmacy, King Saud University, Saudi Arabia). The rats nearly of the same age (12 weeks old) and weighing 220-240 g of King Saud University colony were used. Animals were randomly divided into groups, 12 GNPs-treated rats groups and one control group (NG). Following a period of stabilization (7 days), 10, 20 and 50 nm GNPs were administered intraperitonealy at the rate for 3 or 7 days as follows: Group 1: received infusion of 50 μl GNPs of size 10 nm for 3 or 7 days (n = 10); Group 2: received infusion of 50 μl GNPs of size 20 nm for 3 or 7 days (n = 10); Group 3: received infusion of 50 μl GNPs of size 50 nm for 3 or 7 days (n = 10); Group 4: received infusion of 100 μl GNPs of size 10 nm for 3 or 7 days; (n = 10); Group 5: received infusion of 100 μl GNPs of size 20 nm for 3 or 7 days (n = 10); Group 6: received infusion of 100 μl GNPs of size 50 nm for 3 or 7 days; (n = 10); Control group: received no gold nanoparticles (n = 10).

The rats were maintained on standard laboratory rodent diet pellets and housed in humidity and temperature-controlled ventilated cages on a 12 h day/night cycle. All experiments were conducted in accordance with the guidelines approved by King Saud University Local Animal Care and Use Committee.

Fresh portions of the lateral lobes of the liver from each rat were cut rapidly, fixed in neutral buffered formalin (10%), then dehydrated, with grades of ethanol (70, 80, 90, 95 and 100%). Dehydration was then followed by clearing the samples in 2 changes of xylene. Samples were then impregnated with 2 changes of molten paraffin wax, then embedded and blocked out. Paraffin sections (4-5 um) were stained with hematoxylin and eosin (the conventional histological and stain) according to Pearse [17].

Stained sections of control and treated rats were examined for alterations in the hepatocytes for the presence of inflammatory, fatty change and Kupffer cells hyperplasia and necrosis.

## Results and Discussions

### Size and morphology of different gold nanoparticles

The 10 and 20 nm GNPs show spherical shape while the 50 nm GNPs show hexagonal shape. The mean size for GNPs was calculated from the images taken by the transmission electron microscope (TEM): The 10 nm GNPs was of mean size 9.45 ± 1.33 nm, 20 nm GNPs was of mean size 20.18 ± 1.80 and the 50 nm GNPs was of mean size 50.73 ± 3.58.

### Histological alterations

No mortality occurred in any of the experimental groups of the present investigation, and no alterations were observed in the appearance and behavior of GNPs treated rats in comparison with the control ones.

**Figure 1:**
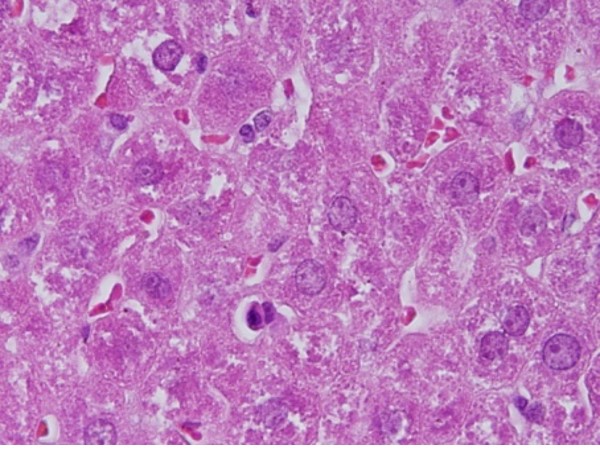
**GNPs-treated rat received 50 μl of 10 nm particles for 3 days demonstrating hepatocytes cloudy swelling**.

In comparison with the control group, the following histological alterations were detected in the liver of GNPs treated rats. These alterations were:

1) Hepatocytes exhibited cloudy swelling with pale cytoplasm and poorly delineated and displaced nuclei in all GNPs treated rats. This ballooning degeneration was more prominent with 100 μl dose than 50 μl one and with 10 nm size particles than the larger ones as shown in Figure 1. This swelling might be exhibited as a result of disturbances of membranes function that lead to massive influx of water and Na^+ ^due to GNPS effects. Cellular swelling might be accompanied by leakage of lysosomal hydrolytic enzymes that lead to cytoplasmic degeneration and macromolecular crowding [18].

The vacuolated swelling of the cytoplasm of the hepatocytes of the GNPs treated rats might indicate acute and subacute liver injury induced by these NPs. Variable nuclei sizes were observed in some hepatocytes. This change became apparent after 7 days of 50 nm GNPs administration.

**Figure 2:**
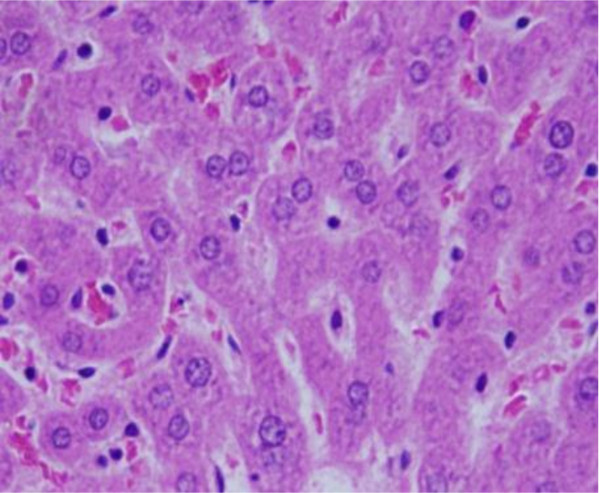
**GNPs-treated rat received 50 μl of 10 nm particles for 7 days demonstrating Kupffer cells hyperplasia**.

**Figure 3:**
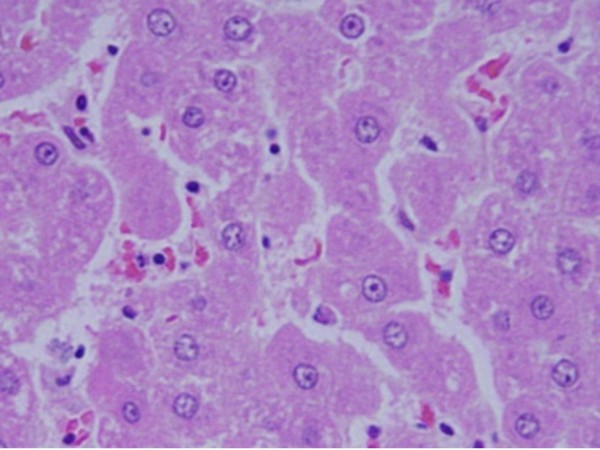
**GNPs-treated rat received 50 μl of 10 nm particles for 3 days demonstrating necrotic hepatocytes**.

**Figure 4:**
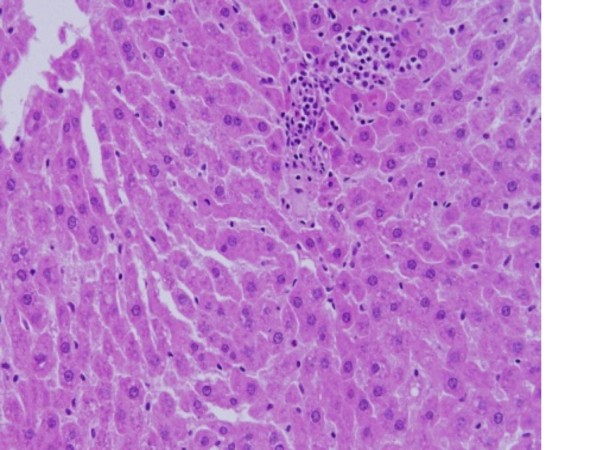
**GNPs-treated rat received 100 μl of 10 nm particles for 3 days demonstrating inflammatory cell infiltration**.

2) The sinusoidal Kupffer cells became prominent and increased in number due to GNPs exposure. This change was more prominent at 10 nm GNPs with dose of 100 μl than 20 nm and 50 nm GNPS and more after 7 days of administration than rats exposed to GNPs for 3 days as shown in Figure 2. Kupffer cells activation might indicate that GNPs activate the phagocytic activity of the sinusoidal cells by increasing the number of Kupffte cells to help in removing the accumulated GNPs where lysosomes are involved in the intracellular breakdown into small metabolic products. The produced Kupffer cells hyperplasia might be correlated with the amount of injurious to the hepatic tissue induced by GNPs intoxication and represents a defense mechanism of detoxification. Kupffer cell hyperplasia is contributed to hepatic oxidative stress [19].

3) Sporadic spotty well-defined necrosis was noticed in some hepatocytes of GNPs treated rats as shown in Figure 3. The insulted cells exhibited highly eosinophilic amorphous cytoplasm with occasional apoptotic characterization. This alteration was detected in the liver of rats exposed to 10 nm size particles and to lesser extent with 20 nm particles but was not seen with those exposed to 50 nm size particles. Apoptic alteration might be followed by organelles swelling, specially mitochondria, endoplasmic reticulum and rupture of lysosomes which might lead to amorphous eosinophilic cytoplasm as an initial sign in the sequence of hepatocytes necrosis before shrinking and dissolution of nuclei [20]. The seen hepatocytes necrosis due to GNPs exposure might indicate oxidative stress on these cells by glutathione depletion.

4) Inflammatory cells infiltration was seen in the portal triads and the perioral zones of GNPs treated rats. The infiltrate cells were mainly lymphocytes and plasma cells as shown in Figure 4. This infiltration was more prominent after 7 days of administration and in rats received 100 μl than those received 50 μl. The appearance of inflammatory cells in the hepatic tissue may suggest that GNPs could interact with proteins and enzymes of the hepatic interstitial tissue interfering with the antioxidant defense mechanism and leading to reactive oxygen species (ROS) generation which in turn may imitate an inflammatory response [21].

GNPs were consistently more strongly oxidizing as evidenced by lipid peroxidation as well as decreased neutral red retention time (NRRT) and numbers of thiol-containing proteins evident in electrophoretic separations. Cadmium may displace iron or copper from metalloproteins leading to oxidative stress via the Fenton reaction [22].

It has been reported that 5 nm GNPs caused significantly greater oxidative stress and cytotoxicity effects than larger ones [23-25]. The 5 nm GNPs have shown to catalyze nitric oxide (NO) production from endogenous S-nitroso adducts with thiol groups in blood serum. NO reacts rapidly with superoxide producing peroxynitrite (ONOO-) which can interact with lipids, DNA, and proteins via direct oxidative reactions or via indirect radical-mediated damage [24]. ROS production could result from the proportionately high surface area of GNPs used in this investigation [26,27].

**Figure 5:**
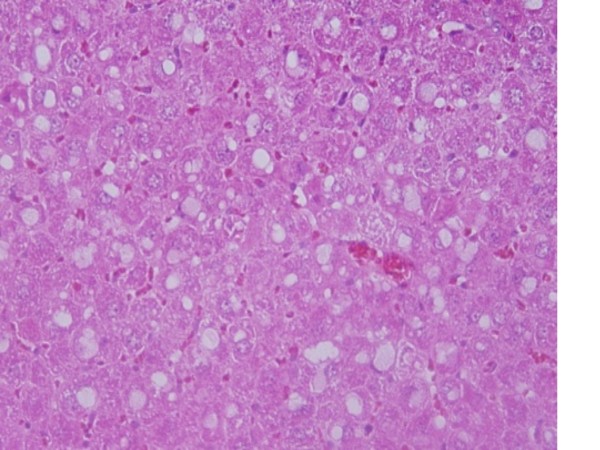
**GNPs-treated rat received 100 μl of 10 nm particles for 7 days demonstrating hepatic fatty degeneration**.

**Figure 6:**
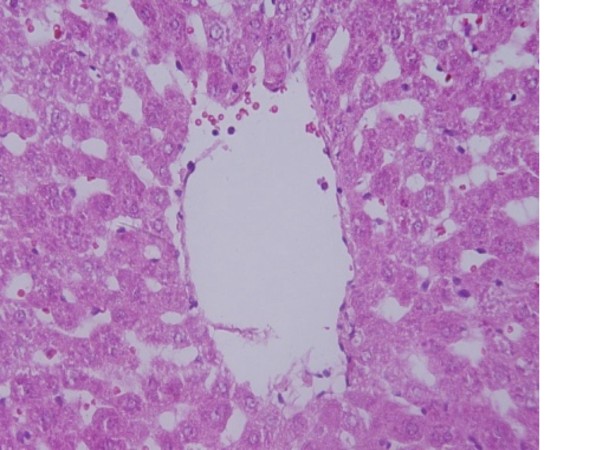
**GNPs-treated rat received 50 μl of 20 nm particles for 7 days demonstrating hepatic central vein intima disruption**.

5) Fatty change was observed in some swelling hepatocytes of rats exposed to 100 μl of 10 nm GNPs and to lesser extent in the ones exposed to larger particles. This hepatic liposis was more prominent in rat exposed to GNPs for 7 days than those received the treatment for 3 days as shown in Figure 5. Hepatocytes fatty change might be due to lipid peroxidation that leads to rough endoplasmic damage and detachment of the cytoplasmic lipoprotein which indicate abnormal fat metabolism [22,25-27]. The seen hepatocytes abnormal retention of lipids in the present investigation induced by GNPs might indicate toxic injury to liver in the form of hepatocytes liposis by these particles.

6) The central veins of the hepatic tissue of rats received 10 and 20 nm GNPs showed disruption of their intima as shown in Figure 6. Less disruption was observed in rats exposed to 50 nm GNPs while more damage was detected after 7 days than 3 days of GNPs exposure. This alteration might indicate endothelial damage and vascular stress by GNPs exposure.

None of the above alterations were observed in the liver of any member of the control group.

Some hepatocytes of rats received 50 nm GNPs showed nucleoli disappearance. This nuclear damage was more prominent after 7 days of exposure to GNPs. Karyorrhexis is a sort of destructive fragmentation of the nucleus processed by pyknosis and followed by karyolysis. Karyolysis is the complete dissolution of the chromatin matter of a dying cell.

Binucleation and to lesser extent polynucleation were observed in GNPs treated rats. Binucleation represents a consequence of cell injury and is a sort of chromosomes hyperplasia which is usually seen in regenerating cells.

**Figure 7:**
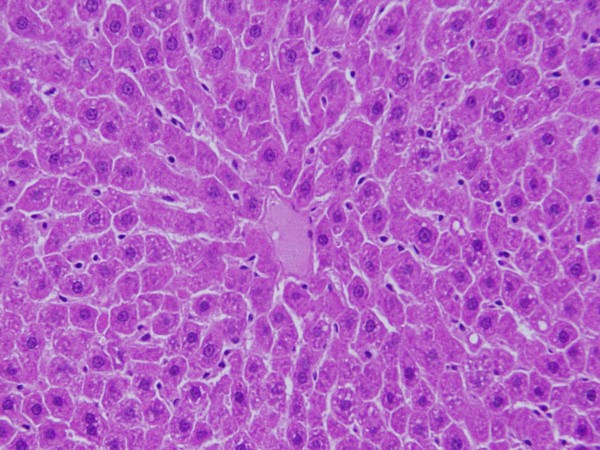
**GNPs-normal rat demonstrating normal hepatocytes**.

The GNPs-normal rat demonstrating normal hepatocytes is shown in Figure 7.

The present study demonstrates that the inflammation produced in the liver and other tissues/or organs was more prominent with smaller GNPs ones induced more affects and related with time exposure of GNPs

Additional experimental investigations related to plasma and tissues (especially liver) cytokine, histomorphologcal and ultrastrucural will be performed to cover and understand the toxicity and the potential use of GNPs as therapeutic and diagnostic tool. It has been reported that smaller GNPs caused significantly greater oxidative stress and cytotoxicity effects than larger ones [23,25].

In present study we have not measured GNPs concentration in urine and feces, but this point will be taken into our consideration in other new additional experiments. It has been reported by Lasagna-Reeves et al., 2010 that administrated NPs were primarily taken up by liver and spleen in a large quantity and small amounts distributed in the lung, kidney, heart, and brain after single administration [26].

## Conclusions

Histological alterations induced by GNPs exposure as shown in the results of the present work could be an indication of injured hepatocytes due to GNPs toxicity that became unable to deal with the accumulated residues resulting from metabolic and structural disturbances caused by these particles. One might conclude that these alterations are size-dependent with smaller ones induced more damage with relation with the time exposure of GNPs.

The appearance of hepatocytes cytoplasmic inflammatory cells infiltration, central vein intima disruption, fatty change and Kupffer cells hyperplasia may suggest that GNPs interact with proteins and enzymes of the hepatic tissue interfering with the antioxidant defense mechanism and leading to reactive oxygen species (ROS) generation which in turn may induce stress in the hepatocytes to undergo necrosis.

Additional supplementary histomorphologcal and ultrastrucural experimental investigations are needed to cover and understand the toxicity and the potential therapeutic and diagnostic use.

## Competing interests

The authors declare that they have no competing interests.

## Authors' contributions

MAKA and BMJ have analyzed data, interpreted and written the final draft of this manuscript. The animal model used in this study was obtained from the Laboratory Animal Center (College of Pharmacy, King Saud University, Saudi Arabia). MAKA has conceived the study and its design and obtained research grants for this study. The authors have read and approved the final manuscript.
